# Nearly 20% of children are not correctly classified according to current ilar classification in a PRINTO dataset of more than 12,000 juvenile idiopathic arthritis patients

**DOI:** 10.1186/1546-0096-12-S1-P176

**Published:** 2014-09-17

**Authors:** Alessandro Consolaro, Francesca Bovis, Ekaterina Alexeeva, Violeta Panaviene, Jordi Anton, Susan Nielsen, Gordana Susic, Maria Trachana, Troels Herlin, Nico Wulffraat, Pavla Dolezalova, Yosef Uziel, Nahid Shafaie, Ingrida Rumba-Rozenfelde, Valda Stanevicha, Nicolino Ruperto, Daniel Lovell, Angelo Ravelli, Alberto Martini

**Affiliations:** 1 Istituto Giannina Gaslini, Genoa, Italy

## Introduction

Juvenile idiopathic arthritis (JIA) is an exclusion diagnosis that encompasses all forms of arthritis that begin before the age of 16 years, persist for more than 6 weeks, and are of unknown origin. In the ILAR classification, this heterogeneous group of chronic arthritides has been categorized on clinical and laboratory grounds to try to identify homogeneous, mutually exclusive categories suitable for etiopathogenic studies. However, the ILAR classification is complex and includes several inclusion and exclusion criteria. As a result, the correct placement of a patient in a specific category is not simple.

## Objectives

To assess the rate of inappropriate classification in a large dataset of JIA patients collected by PRINTO members.

## Methods

Patients enrolled in the multinational study of the EPidemiology, treatment and Outcome of Childhood Arthritis (EPOCA study) and in the Pharmacovigilance in patients treated with biologics± methotrexate study (Pharmachild) were merged in a single database, after exclusion of overlapping patients. The reasons that led to a “provisional” ILAR classification (i.e. lack of fitting into an ILAR category despite ILAR category attribution by the attending physician) in the two datasets and the queries regarding classification raised to the investigators by the PRINTO staff were analyzed and grouped into major categories according to the inclusion or exclusion criterion involved.

## Results

A total of 12,141 patients were included in the study. The Table shows, for each JIA subtype, the most frequent drawbacks leading to a provisional classification. Most problems were related to the lack of 2 determinations of rheumatoid factor (RF) at least 3 months apart, the missing data in the indication of the presence or absence of psoriasis in the patient or in the presence or absence of a history of psoriasis in a first degree relative, the lack of assessment of HLA-B27 antigen, or the discrepancies in data results in the indication of a family history of spondyloarthropathies.

**Table 1:** 

	N	Provisional diagnosis N (%)	Reasons for provisional diagnosis N (%)			
			
			Rheumatoid factor	Psoriasis	Spondylitis features	HLA-B27
**Systemic arthritis**	1365	295 (21.6)	219 (74.2)	83 (28.1)	67 (22.7)	34 (11.5)

**Oligoarthritis**	4887	1127 (23.1)	837 (74.3)	353 (31.3)	314 (27.9)	144 (12.8)

**Polyarthritis RF-negative**	2991	379 (12.7)	273 (72)	183 (48.3)	157 (41.4)	69 (18.2)

**Polyarthritis RF-positive**	492	277 (56.3)	266 (96)	33 (11.9)	28 (10.1)	10 (3.6)

**Psoriatic arthritis**	433	101 (23.3)	63 (62.4)	22 (21.8)	23 (22.8)	7 (6.9)

**Enthesitis related arthritis**	1323	217 (16.4)	118 (54.4)	90 (41.5)	-	-

**Total**	12141	2396 (19.7)	1776 (74.1)	764 (31.9)	589 (24.6)	264 (11)

## Conclusion

In current clinical practice nearly 20% of JIA patient were categorized according to physician diagnosis attribution despite the lack of fulfillment of the ILAR exclusion criteria. Most frequently, this was related to the lack of assessment of RF or the inconsistency in indication of the presence of psoriasis in a first-degree relative.

## Disclosure of interest

None declared.

